# Assessment of vitamin A, vitamin B_2_, vitamin B_12_, vitamin K, folate, and choline status following 4 months of multinutrient supplementation in healthy vegans: a randomised, double-blind, placebo-controlled trial

**DOI:** 10.1007/s00394-025-03814-7

**Published:** 2025-12-19

**Authors:** Tim Zerback, Christian Koeder, Stine Weder, Andreas Sputtek, Gunter P. Eckert, Markus Keller

**Affiliations:** 1 Institute for Prevention and Cancer Epidemiology (IPE), Medical Center-University of Freiburg, Faculty of Medicine, University of Freiburg, 79110 Freiburg im Breisgau, Germany; 2 Research Institute for Plant-Based Nutrition, 35444 Biebertal, Germany; 3 MVZ Medical Laboratory Bremen GmbH, 28359 Bremen, Germany; 4 Institute of Nutritional Sciences, Justus-Liebig University, 35392 Giessen, Germany

**Keywords:** Vegan diet, Riboflavin, Multinutrient, Vitamin K_2_, Vitamin A, Cobalamin

## Abstract

**Purpose:**

The aim of the MultiVeg study, a double-blind, randomised controlled trial (RCT), was to investigate the nutritional status of healthy vegans following 4 months of multinutrient supplementation.

**Methods:**

A double-blind, RCT was conducted with 72 vegan adults (19–57 years) in Germany. Data on anthropometric parameters, dietary nutrient intake, and nutritional status were collected. The nutritional status of the participants was assessed at baseline and after 4 months. The results were compared between groups using ANCOVA. The results for vitamins and choline are presented here.

**Results:**

After adjustment for baseline values, age, sex, and multiple testing, no significant between-group differences in biomarker concentration changes from baseline to 4 months were observed for vitamin A, retinol-binding protein, transthyretin, beta-carotene, methylmalonic acid, homocysteine, choline, total osteocalcin, carboxylated and undercarboxylated osteocalcin, and folate. In contrast, significant between-group differences in changes were observed for flavin adenine dinucleotide (FAD), serum vitamin B_12_, holotranscobalamin, and the combined vitamin B_12_ status indicator (cB_12_) after adjustment.

**Conclusion:**

A multinutrient supplement containing 82 µg of vitamin B_12_ per day significantly positively affected vitamin B_12_ blood biomarkers in healthy vegans.

**Registration:**

This study was registered in the German Clinical Trials Register (DRKS00028151).

**Supplementary Information:**

The online version contains supplementary material available at 10.1007/s00394-025-03814-7.

## Introduction

Vegan diets have gained increasing popularity in many countries around the world. A recent review estimated the percentage of adults calling themselves vegan in Germany to be approximately 3% [1]. For vegan diets, several nutrients may be considered potentially critical such as vitamin B_12_, vitamin D, vitamin B_2_, iron, zinc, iodine, and selenium [2]. Furthermore, since conversion rates from α-linolenic acid to docosahexaenoic acid (DHA) are estimated to typically be low, beneficial effects of a direct intake of the long-chain omega-3 fatty acids eicosapentaenoic acid (EPA) and DHA for vegans are being discussed [3]. In addition, vitamin A is currently under discussion as a potentially critical nutrient in vegan diets [4]. Humans can obtain vitamin A in two ways: as preformed vitamin A (retinol or other retinoids) from animal products or supplements and as provitamin A (beta-carotene and other provitamin A carotenoids) from plants or other non-animal foods [5]. However, genetic polymorphisms in the beta-carotene 15,15’-monooxygenase (BCMO1) gene, the key enzyme responsible for converting beta-carotene into retinal, may potentially lead to an insufficient vitamin A status [6, 7], especially in vegans. In a small study with young, non-obese women, approximately 27–45% exhibited such genetic dispositions resulting in a 32–69% decreased beta-carotene conversion [8]. Affected individuals may hardly convert beta-carotene to vitamin A, leading to a vitamin A deficiency and hypercarotenaemia, if they adhere to a retinol-poor diet such as a vegan diet [9]. These low-converters, if following a vegan diet, might benefit from supplementation with retinol at doses of 500–1000 µg per day without exceeding the recommended dosage [10]. Moreover, the bioavailability of vitamin A depends on several factors, including intake of retinol and beta-carotene, concomitant fat ingestion, genetic factors, and food preparation techniques [11]. For vitamin B_12_, there is a consensus among nutrition scientists that this vitamin should be supplemented when adhering to a vegan or near-vegan diet unless an adequate intake of this vitamin is ensured via fortified foods [12]. Apart from vitamin B_12_, vegans may benefit from supplementing other nutrients like vitamin B_2_, vitamin D, calcium, iron, iodine and selenium, depending on which vegan foods are habitually consumed, whether fortified products are chosen, as well as other circumstances such as sun exposure (for endogenous vitamin D synthesis) [2]. The primary source of vitamin B_2_ (riboflavin) in mixed diets are dairy products [13]. It is therefore considered a potentially critical nutrient in vegan diets, a view supported by the evidence that vegans often had the lowest vitamin B_2_ status in studies, compared to vegetarians and omnivores [2, 14, 15]. Finally, choline is also discussed as a potentially critical nutrient in vegan diets as its primary sources are animal-based foods like eggs, poultry, and meat [16, 17].

Currently, a variety of nutrient supplements specifically designed for vegans are available on the market. While a small multinutrient intervention study with vegans was conducted in the 1960s, to date [18], no randomised controlled trial (RCT) appears to have assessed the respective nutrient status of vegans before and after 4 months of multinutrient supplementation.

Therefore, it was the aim of the present study to assess the status of vitamin A and vitamin B_12_ as well as vitamin B_2_, vitamin K, folate, and choline in healthy vegans before and after 4 months of supplementation of a multinutrient supplement. The supplement included three distinct products: a multinutrient capsule containing vitamin A, vitamin B_2_, vitamin B_12_, vitamin D_3_, vitamin K_2_, calcium, iodine, iron, selenium, and zinc; an omega-3 supplement (providing EPA, DHA, vitamin D_3_, and vitamin E); and a soya lecithin powder rich in choline. Additionally, we did a subgroup analysis (e.g., single vs. double dosage in the omega-3 supplement and separate reference values for ferritin levels in men and women). Examination of mineral constituents within the multinutrient supplement as well as the omega-3 supplement (containing vitamins D_3_ and E) is outside of the scope of this analysis and will be published elsewhere.

## Subjects and methods

### Study design

The MultiVeg study is a single-centre (Bad Homburg), double-blind, RCT collecting data on anthropometric parameters, dietary nutrient intake, and nutritional status of vegan adults in Germany from October 2022 (baseline, t0) to February 2023 (4-month follow-up, t2).

Participants in the intervention group (INT) were instructed to take a multinutrient supplement (1 capsule daily), an omega-3 supplement (2 capsules daily, also containing vitamin D_3_ and vitamin E) as well as a supplement in powder form (choline: ~320 mg/day) for 4 months, preferably with a meal. The control group (CON) received placebo supplements, which had the same organoleptic properties. The objective of this analysis is to assess the effect of the multinutrient supplement on blood biomarkers for vitamins and choline. Both groups were instructed to maintain their regular dietary habits throughout the intervention. Additionally, all supplements underwent laboratory analysis performed by an external laboratory (Gesellschaft für Bioanalytik mbH, Hamburg, Germany) to determine their nutrient content (active compounds) or lack thereof (placebo) (Tables 1, 2 and 3). Details regarding randomisation and group allocation can be found in Supplementary Fig. 1.

Compliance, defined as taking the study supplements exactly as instructed, was assessed based on self-reported data at t2.

The study was conducted according to the guidelines of the Declaration of Helsinki and was approved by the ethics committee of the Faculty of Medicine of the University of Giessen on June 28, 2022 (reference: 57/22).

**Table 1:** Declared and actual composition of the vegan multinutrient supplement (vitamins only, daily dose: 1 capsule)

Nutrient	Declared nutrient content (per capsule)	Actual nutrient content^a^ (per capsule)
Vitamin A (retinyl acetate and retinyl palmitate, 1:1 ratio)	500 µg	743 µg
Vitamin B_2_ (sodium-riboflavin-5-phosphate)	1.40 mg	1.03 mg
Vitamin B_12_ (methyl-, hydroxo-, and adenosylcobalamin, 1:1:1 ratio)	100 µg	82 µg
Vitamin D_3_ (cholecalciferol)	25 µg	26 µg
Vitamin K_2_ (all-trans menaquinone-7)	50 µg	45 µg
Calcium (calcium bisglycinate)	120 mg	129 mg
Iodine (potassium iodide)	150 µg	154 µg
Iron (iron bisglycinate)	6.0 mg	7.2 mg
Selenium (sodium selenite)	55 µg	70 µg
Zinc (zinc bisglycinate)	6.5 mg	6.4 mg

^a^ According to laboratory analysis (GBA Gesellschaft für Bioanalytik mbH, Hamburg)

**Table 2:** Declared and actual composition of the vegan omega-3 supplement (daily dose: 2 capsules)–not objective of this Article

Nutrient	Declared nutrient content (per capsule)	Actual nutrient content^a^ (per capsule)
EPA	At least 75.0 mg	98.7 mg
DHA	At least 150 mg	171 mg
Vitamin D_3_ (cholecalciferol)	25 µg	36 µg
Vitamin E (d-α, d-β, d-γ- & d-δ tocopherol)	3.0 mg	3.7 mg

EPA: eicosapentaenoic acid; DHA: docosahexaenoic acid

^a^ According to laboratory analysis (GBA Gesellschaft für Bioanalytik mbH, Hamburg)

**Table 3:** Declared and actual composition of the vegan choline supplement (daily dose: 11.5 g, equivalent to one level measuring spoon)

Nutrient	Declared nutrient content	Actual nutrient content^a^
Soya lecithin	11.5 g	–
of which phosphatidylcholine	2.30 g	2.07 g
of which choline	300.0 mg	321.5 mg

^a^ According to laboratory analysis (GBA Gesellschaft für Bioanalytik mbH, Hamburg)

### Recruitment

**Fig. 1:**
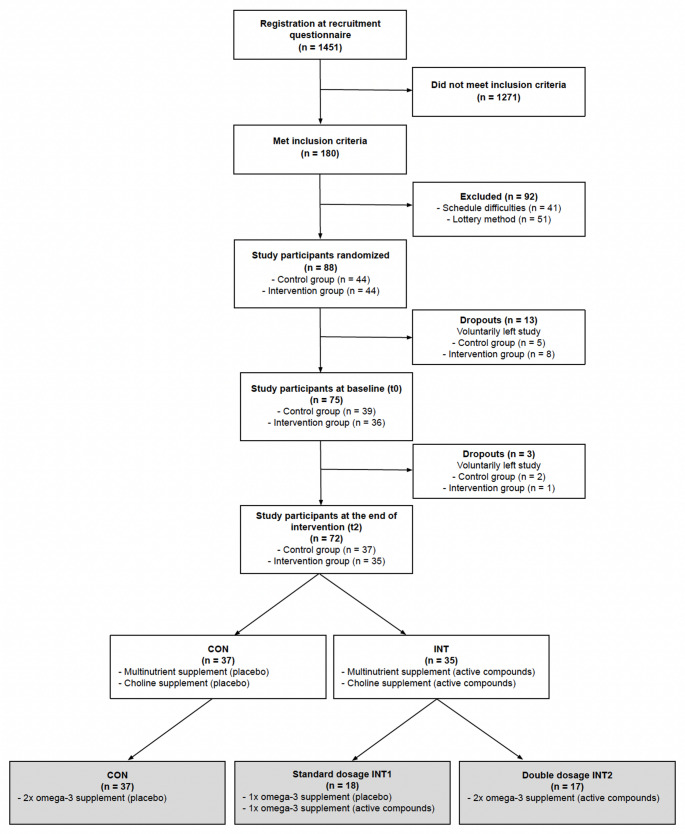
Flow chart of participants through the study with randomisation and group allocation in detail CON: control group; INT: intervention group; INT1: intervention group standard dosage; INT2: intervention group double dosage (the only vitamins affected by this subgroup intervention were vitamins D and E). Cells marked in grey indicate differences in the intake of the omega-3 supplement, which is the primary focus of another paper.

Vegan subjects were recruited in August 2022 through various online channels including YouTube, the Research Institute for Plant-Based Nutrition participant database, newsletters, and social media platforms. Interested individuals completed an online recruitment questionnaire and were informed of the inclusion and exclusion criteria prior to their participation. Eligible participants were then selected based on inclusion and exclusion criteria (Fig. 1).

Inclusion criteria: Participants were required to be ≥ 18 years of age and to have followed a vegan diet (self-reported and verified by questions about frequency of consumption of animal foods), defined as the exclusion of meat, fish/seafood, dairy products, and eggs for ≥ 1 year. However, it was allowed and accepted if participants reported consuming meat and/or meat products and fish and/or fish products less than once a month, and dairy products and/or eggs and/or egg products (including those in bakery products, pasta or processed foods) no more than 3 times a month.

Exclusion criteria: Not accepted were smoking, pregnancy, lactation, diagnosis of any vitamin or mineral deficiency by a physician, a chronic medical condition (e.g., chronic gastritis, chronic gastrointestinal disease, thyroid disorders, or other absorption-related disorders), or regular use of medication (except contraceptive drugs). Additionally, participants were excluded if they had engaged in any of the following within three months prior to the study commencement:

- supplementation of vitamin A, beta-carotene, or selenium (any amount)
- average intake of > 2 Brazil nuts per day
- vitamin B_12_ supplementation of > 1000 µg once per month, or > 200 µg once a week, or > 50 µg once per day, or use of vitamin B_12_-fortified toothpaste more than once per day
- supplementation of vitamin B_2_, vitamin D, vitamin E, zinc, iron, choline, or omega-3 fatty acids more than once a week (any amount)
- regular supplementation of iodine (any amount; iodised salt was permitted) or consumption of iodine-rich seaweed more than once a week (permitted: up to 1 teaspoon of nori flakes per day or up to 1 nori sheet per day).

To prevent any interference with the investigated multinutrient supplement, preference was given to participants taking few or no dietary supplements.

### Study schedule

If study inclusion criteria were met, participants received detailed written information about the study. 180 individuals met the inclusion criteria. The individuals were contacted for scheduling appointments at the study center (t0, t2). To initially allocate 96 appointment slots, a randomised approach with a lottery method (51 were excluded) was adopted, aiming for a balanced distribution (1:1) of male and female participants. Due to scheduling difficulties, 41 could not participate (Fig. 1).

All participants gave their written informed consent prior to inclusion in the study. Participants (*n* = 88) were allocated to either the INT or the CON using the Clinical Trial Randomisation Tool by the National Cancer Institute (USA; https://ctrandomization.cancer.gov/tool/). Disclosure of group allocation to the investigator who conducted the primary statistical analysis (T.Z.) as well as to study participants was performed only after statistical analysis had been conducted.

For full study participation, the study subjects received an incentive of 100 Euros each (at t2) and were provided with their laboratory results.

### Anthropometric measurements

Anthropometric measurements were performed in the fasted state and by trained staff. Body weight was assessed with the participants in underwear, without shoes, using scales (Seca 799, Hamburg, Germany). Height was assessed using a stadiometer (Seca 222).

### Biomarker selection

As one of the main outcomes, for vitamin A four biomarkers were measured: Firstly, serum retinol as primary marker of vitamin A status reflecting recent intake [19]. Secondly, retinol binding protein (RBP) as a sensitive indicator because it transports retinol and decreases in vitamin A deficiency states. Thirdly, transthyretin (TTR) assists in retinol transport and reflects overall vitamin A status [20, 21]. Finally, beta-carotene, a precursor of vitamin A, is influenced by diet and conversion efficiency, making it a variable marker [19]. As a second main outcome, for vitamin B_12_ also four parameters were investigated. Serum vitamin B_12_ is the most commonly used marker to assess vitamin B_12_ status, but it may lack sensitivity, particularly in cases of subclinical deficiency. In contrast, holotranscobalamin (holoTC) is considered a more sensitive marker as it represents the fraction of vitamin B_12_ available for cellular uptake. Additionally, methylmalonic acid (MMA) is a functional marker that specifically increases in vitamin B_12_ deficiency, reflecting impaired enzyme activity in vitamin B_12_-dependent pathways. Finally, homocysteine (Hcy) levels also increase with vitamin B_12_ deficiency, although they are less specific as they can be influenced by folate and other factors [22]. In combination, these four parameters allow a more accurate determination of vitamin B_12_status, particularly when using the combined indicator cB_12_. This approach integrates serum vitamin B_12_, holoTC, MMA, and Hcy into a single diagnostic value, expressed as cB_12_ = log10[(holoTC × B_12_) / (MMA × tHcy)] – (age factor). This formula enhances the accuracy of deficiency diagnosis by accounting for the interactions between these biomarkers [23]. For vitamin B_2_, flavin adenine dinucleotide (FAD), a coenzyme form of vitamin B_2_ that reflects the functional status of this vitamin at the cellular level, was determined [24]. To evaluate the interaction of folate and vitamin B_12_ with homocysteine metabolism, whole blood folate was analysed. Whole blood folate levels provide a stable and accurate measure of long-term folate status, reflecting folate stores in tissues over several months, making it a superior indicator compared to serum or plasma folate, which only reflects recent dietary intake [25, 26]. For choline, there is currently a lack of highly reliable biomarkers for assessing its status. Plasma choline is the most commonly used marker, but has significant limitations in that its levels do not consistently correlate with dietary intake or tissue stores [27]. Despite these limitations, plasma choline is still employed due to the absence of superior alternatives [28]. Furthermore, vitamin K-related bone health markers, such as osteocalcin (OC), were also measured. OC is a vitamin K-dependent calcium-binding protein that strongly binds to hydroxyapatite. OC is metabolically activated to its functional, carboxylated form (cOC). Under conditions of vitamin K deficiency and/or increased bone turnover, undercarboxylated osteocalcin (ucOC) is elevated in blood [29].

### Blood samples

Venous blood samples were drawn in the morning after an overnight fast. To verify fasting, participants were asked about the last time they had eaten or consumed caloric beverages. With the exception of carboxylated and undercarboxylated osteocalcin, all blood parameters were analysed at MVZ Medical Laboratory Bremen GmbH. Serum samples were obtained by centrifugation at 2500 x g for 10 min within 1 h after blood sampling. Whole blood and serum samples were stored light-protected and frozen at − 20 °C for a maximum of 3 months and shipped to the laboratory on dry ice. Vitamin B_2_ and folate were determined in EDTA whole blood, choline in EDTA plasma. Vitamin B_12_, holoTC, MMA, Hcy, vitamin A, RBP, TTR, beta-carotene, and total OC were determined in serum. cOC and ucOC in serum samples were assessed using enzyme immunoassay (EIA) kits (Takara Bio Inc, Shiga, Japan) at BioTeSys–Nutritional CRO & Testing Laboratory GmbH, Esslingen. For the measurement of FAD, an isocratic HPLC in-house method with fluorimetric detection was applied. Vitamin B_12_, OC, and folate were assessed with electrochemiluminescence immunoassays (Cobas, Roche Diagnostics, Mannheim, Germany). An enzymatic immunoassay (IBL International, Hamburg, Germany) was used for the measurement of holoTC, and a gas chromatographic in-house method (non-polar capillary separation column with single-ion monitoring detection) was used for MMA. Hcy and choline were determined by means of an in-house HPLC-tandem mass spectrometry method. For the measurement of the primary marker, vitamin A and beta-carotene, isocratic in-house HPLC-methods with UV (313 nm) and spectrophotometric detection (460 nm), respectively, were used. Turbidimetric immunoassays on an Optilite (The Binding Site, Schwetzingen, Germany) were used for the measurement of RBP (Trimero Diagnostics, Barcelona, Spain) and TTR (The Binding Site). The day-to-day coefficients of the analytical methods varied from 1–10%, depending on the method. The laboratory reference values used to evaluate nutrient status are given in Table 4.

### Dietary assessment

Participants completed a 3-day weighed dietary record in the period of one week before to one week after the respective examination time points (t0, t2). These two dietary protocols were used to adjust for the mean intake values of each nutrient, measured as %DACH (individually achieved percentage of the dietary reference values for Germany, Austria and Switzerland [D-A-CH]) [30]. Additionally, a 3-day weighed dietary record at 2 months (t1) during the midpoint of the intervention was also completed by the participants. This dietary food record at t1 was primarily used to monitor any changes in dietary habits during the 4-month intervention period and to identify any potential deviations in nutrient intake for subsequent discussion.

All foods and beverages consumed as well as leftovers were weighed and recorded over 3 days using electronic kitchen scales. Participants also collected the packaging of convenience food products which they had consumed. When exact weighing was not possible (for example, in the case of eating outside or regarding table salt and spices), semi-quantitative household measurements (e.g., tablespoons, teaspoons, or cups) or photos were permitted for determination of portion sizes. For each time point, energy and nutrient intakes were calculated as the mean intake of the 3 days reported. The analysis of dietary nutrient intake data was conducted using the nutritional analysis software Optidiet Basic (Version 6.0.0.001, GOE GmbH, Linden, Germany). The nutrient composition of foods was based on the German Nutrient Database (Bundeslebensmittelschlüssel, BLS, version 3.02). Energy and nutrient content of convenience food products (ready-to-eat meals or snack foods) were estimated by recipe. Simulation was conducted using labelled ingredients and nutrient contents, including those added through fortification.

Choline intake was not assessed as the German Nutrient Database (BLS 3.02) does not contain data regarding choline content of foods.

**Table 4:** Examined nutrient biomarkers and the laboratory’s reference ranges

Nutrient	Nutrient biomarkers [unit]	Reference ranges
Vitamin A	Vitamin A [mg/L]	0.2–1.2 [31]
	RBP [mg/dL]	2.08–4.25^a^
	TTR [g/L]	0.20–0.40 [32]
	Beta-carotene [µg/L]	150–1250^b^
Vitamin B_2_	FAD [µg/L]	199–382 [24]
Vitamin B_12_	Vitamin B_12_ [pg/mL]	197–771 [33]
	HoloTC [pmol/L]	21–123^c^ [33]
	MMA [µg/L]	9–32 [33]
	Hcy [µmol/L]	< 13 [33]
	cB_12_	− 0.5–1.5^d^ [23]
Choline	Choline [µg/L]	728–1287 [34]
Folate	Folate [µg/L]	212–534 [35]
Vitamin K	OC [ng/mL]	11–70^e^
	cOC [ng/mL]	no reference range available
	ucOC [ng/mL]	no reference range available

RBP: retinol-binding protein; TTR: transthyretin; FAD: flavin adenine dinucleotide; HoloTC: holotranscobalamin; MMA: methylmalonic acid; Hcy: homocysteine; cB_12_: combined indicator for vitamin B_12_ (calculated); OC: total osteocalcin; cOC: carboxylated osteocalcin; ucOC: undercarboxylated osteocalcin

^a^ CE-certified test, according to test manufacturer (Trimero Diagnostics, Barcelona, Spain)

^b^ From measurements in healthy volunteers, *n* = 30, Medical Laboratory Bremen

^c^ <35: vitamin B_12_ deficiency is likely, 35–50: vitamin B_12_ deficiency is possible, >50: vitamin B_12_ deficiency is unlikely [33]

^d^ >1.5: elevated vitamin B_12_, − 0.5–1.5: vitamin B_12_ adequacy, − 1.5– − 0.5: low vitamin B_12_, − 2.5– − 0.5: possible vitamin B_12_ deficiency, <− 2.5: probable vitamin B_12_ deficiency [23]

^e^ CE-certified test, according to test manufacturer (Roche Diagnostics, Mannheim, Germany)

### Statistics

The sample size was calculated based on the primary parameter of the study (vitamin A) using the program G*Power (version 3.1.9.2). To our knowledge, no other study has compared vegans who supplemented vitamin A to vegans who did not supplement vitamin A. Therefore, the calculation was based on a study from Switzerland (*n* = 206) which investigated serum retinol levels in vegans and omnivores. For the calculation, a difference in serum retinol levels between omnivores (1869 ± 436 nmol/L) and vegans (1562 ± 408 nmol/L) was found to be 307 nmol/L [15]. Based on this assumption, a strong effect size (Cohen’s d) of approximately 0.7 was anticipated, and the required number of participants was determined to be 31 per study group, resulting in a power of 0.8 and a two-sided α of 0.05 (a priori two-sided t-test). Overall, a dropout rate of 30–40% (based on a previous study [36]) was expected. Thus, a total sample size of 44 per group was aimed for.

For continuous variables, baseline characteristics are presented as means ± standard deviation (SD) or median and interquartile range (IQR). Categorical variables are presented as numbers and percentages.

Shapiro-Wilk test was employed to test for non-normality in the data. Fisher’s exact test was utilised for comparing categorical variables, while independent t-test was applied to normally distributed, continuous variables and Mann-Whitney U test for those that were non-normally distributed (two-sided tests). To evaluate within-group changes in blood parameters, paired t-test was used for normally distributed and Wilcoxon test was used for non-normally distributed data (two-sided tests).

To assess differences in blood parameters between the two groups, independent t-test was employed for normally distributed data while Mann-Whitney U test was utilised for non-normally distributed data (two-sided tests). To assess intragroup differences, a paired samples t-test was employed for data that were normally distributed, while the Wilcoxon test was utilised for non-normally distributed data (two-sided tests). In addition, one-way analysis of covariance (ANCOVA) was performed, adjusting for baseline values [37]. ANCOVA analyses were repeated adjusting for other potential confounders (age, sex, and each nutrient’s intake). For dietary intake adjustments, the mean intake was calculated from the available records at both time points (t0 and t2). If one of these records was missing (*n* = 6), the mean intake was based on a single time point (t0 or t2).

Statistical significance was set at the 0.05 level. Bonferroni-Holm correction was applied to control for multiple testing [38]. All analyses were conducted using IBM SPSS Statistics (Version 28.0, Armonk, NY).

## Results

For 72 participants blood values were available for both time points (t0, t2). These participants were included in the analysis. Of these, only 66 provided both 3-day weighed dietary records, while 67 participants submitted a mid-term dietary record at t1 (Table 6). Figure 1 shows trial details.

### Baseline characteristics

No significant differences were observed between the two groups in terms of baseline characteristics such as sex, age, body weight, height, BMI, and duration of adherence to a vegan diet (Table 5).

Some participants reported occasional consumption of animal products or supplements in the recruitment questionnaire (Supplementary Table S2).

Only a small percentage reported taking any supplements within the study’s allowed limits (CON 18.9%, INT 20.0%), with most of them supplementing vitamin B_12_ (CON 13.5%, INT 14.3%), vitamin D_3_ (CON 8.1%, INT 5.7%), vitamin K_2_ (CON 2.7%, INT 5.7%), iron (CON 2.7%, INT 0.0%), and omega-3 (CON 2.7%, INT 0.0%).

**Table 5:** Baseline characteristics of the MultiVeg study participants

Variables [unit]	CON (*n* = 37)	INT (*n* = 35)	*p*-value^a^
Men [n (%)]	18 (48.6)	19 (54.3)	1
Age at baseline [years]^b^	26.0 (10.0)	25.0 (7.0)	1
Body weight [kg]^c^	68.8 ± 12.0	68.0 ± 11.3	1
Height [cm]^c^	175.1 ± 9.2	175.9 ± 9.5	1
BMI [kg/m^2^]^c^	22.4 ± 3.7	21.9 ± 2.9	1
Vegan diet duration [years]^b^	3.5 (2.4)	2.5 (3.5)	1

BMI: body mass index

^a^ Fisher’s exact test (categorical variables), independent t-test (normally distributed continuous variables), or Mann-Whitney U test (non-normally distributed continuous variables). P-Value are adjusted for multiple testing by Bonferroni-Holm correction

^b^ Median (IQR)

^c^ Mean ± SD

### Compliance

In the INT, compliance was relatively high (71.4% for daily intake, according to [39]) for the multinutrient supplement. The remaining subjects followed the intake instructions six (22.9%) or five (5.7%) times a week. Compliance regarding the choline supplement (powder) was moderate: only 19 of 35 participants (54.3%) adhered to the daily intake instructions, eleven (31.4%) took it six times a week, three (8.6%) five times a week, and the remaining two participants (5.7%) only three times a week.

In the CON, compliance was comparable: 27 of 37 participants (73.0%) consumed the placebo daily, as instructed. The remaining subjects took it six (24.3%) or five (2.7%) times a week. For choline, 22 out of 35 participants (59.5%) adhered to the daily intake instructions, five (13.5%) adhered six times a week, eight (21.6%) five times a week, one (2.7%) four times a week, and one participant (2.7%) adhered three times a week.

### Dietary vitamin intake (including fortified foods and supplements, excluding the supplements that were part of the study) at baseline (t0), after 2 months (t1) and after 4 months (t2).

Median dietary vitamin A intake (calculated as retinol equivalent, RE) was between 64.8 and 82.9% of the dietary reference values (DRV), expressed as %DACH (individually achieved proportion of the DRV for Germany, Austria and Switzerland). Median dietary vitamin B_2_ intake was between 64.5–99.6%DACH in both groups at all three time points. In both groups and at all three time points, median dietary vitamin B_12_ intake was ≤ 12.9%DACH.

No significant differences in dietary vitamin intake between the INT and CON were observed at any of the time points. Median dietary folate intake, calculated as dietary folate equivalent (DFE), was only slightly below the DRV in the INT at t2 (97.6%DACH) while the INT and CON met the DRV at all other time points.

Median dietary nutrient intakes of vitamin K exceeded the dietary reference values in both the INT and CON at all three time points (t0, t1, and t2) (Table 6).

**Table 6:** Median (IQR) nutrient intake (including fortification and supplements, excluding the supplements that were part of the study) of the participants of the MultiVeg study at baseline (t0), after 2 months (t1), and after 4 months (t2), measured as %DACH (individually achieved proportion of the DRV for Germany, Austria and Switzerland) [30]

Nutrients (%DACH)	CON (*n* = 33)^a^			INT			*p*-values^b^		
	t0	t1	t2	t0 (*n* = 33)^a^	t1 (*n* = 34)^a^	t2 (*n* = 33)^a^	t0	t1	t2
Vitamin A (RE)^c^	82.9 (73.8)	77.9 (93.9)	74.2 (69.4)	75.7 (89.1)	57.0 (54.9)	64.8 (71.9)	1	1	1
Vitamin B_2_^c^	99.6 (62.3)	86.7 (49.6)	92.6 (42.0)	77.3 (50.5)	64.5 (38.3)	76.1 (39.0)	1	0.582	0.855
Vitamin B_12_^c^	12.9 (26.3)	11.3 (23.7)	8.9 (19.2)	10.1 (28.1)	2.3 (13.5)	6.7 (13.5)	1	1	1
Choline	NA	NA	NA	NA	NA	NA	NA	NA	NA
Folate (DFE)^c^	126.9 (88.9)	118.1 (58.0)	110.1 (58.4)	113.4 (70.1)	102.8 (62.0)	97.6 (44.6)	1	1	1
Vitamin K^c^	318.1 (345.0)	357.4 (379.3)	291.7 (297.1)	311.4 (272.7)	243.6 (343.7)	228.6 (220.5)	1	1	1

^a^ Missing at t0 and t2: *n* = 4 (CON), *n* = 2 (INT). Missing at t1: *n* = 4 (CON), *n* = 1 (INT) ^b^ Mann-Whitney U test (non-normally distributed continuous variables) for between-group differences, adjusted by Bonferroni-Holm correction. ^c^ Median (IQR). NA = Choline intake calculation could not be performed, as there are no food composition data available for choline in the German Nutrient Database (Bundeslebensmittelschlüssel BLS 3.02). RE = retinol equivalent. DFE = dietary folate equivalent

### Percentage of participants with circulating biomarkers outside of the reference ranges

At baseline, most of the participants (> 75%) of each group were within or above the reference ranges (for Hcy and MMA below or within the recommended value[s]) for most nutrient biomarkers, except for RBP (40% [CON], 46% [INT]), and for choline (57% [CON], 54% [INT]). At t2, most participants (> 75%) in each group still fell within or exceeded the reference ranges for most nutrients. However, a proportion of participants had values below the reference ranges, notably for RBP (76% [CON], 60% [INT]) and for TTR (30% [CON]). Details are shown in Supplementary Table S1.

### Vitamin status at baseline (t0) and after 4 months (t2)

For all biomarkers of vitamin status, no significant differences between the two groups (intergroup differences) were observed at t0 and t2 (Table 7). Except for RBP and FAD, mean or median values for each of the other nutrient biomarkers assessed were within the reference ranges at t0 and t2 (Table 4). For RBP, the median levels in both groups were below the reference range (2.08–4.25 mg/dL) at both time points (indicating low vitamin A status). For FAD at t2, the mean value was below the reference range (199–382 µg/L) only in the CON.

Additionally, significant intragroup differences (t0 vs. t2) were observed for nutrient biomarkers in both groups except for beta-carotene, holoTC, Hcy, cB_12_, and cOC in the CON, and RBP, TTR, beta-carotene, vitamin B_12_, Hcy, folate, and cOC in the INT (Supplementary Table S3).

### Changes in vitamin status from baseline (t0) to 4 months (t2)

No significant between-group differences in biomarker concentration changes from baseline to 4 months were observed for vitamin A, RBP, TTR, beta-carotene, MMA, Hcy, choline, OC, cOC, ucOC, and folate. In contrast, significant between-group differences in changes were detected for all other nutrient biomarkers analysed (Table 7) after adjusting for baseline, age, and sex: FAD − 21 [95% confidence interval: − 29; − 13] µg/L, vitamin B_12_ − 90 [− 134; − 45] pg/mL, holoTC − 14 [− 18; − 9] pmol/L, cB_12_ − 0.42 [− 0.57; − 0.27]. For MMA, the between-group difference was 5 [2; 7] µg/L. The difference in mean changes in MMA between the groups was statistically significant only in the baseline-adjusted analysis, with a* p*-value of 0.048.

**Table 7:** Average nutrient biomarkers concentration at baseline (t0) and after 4 months (t2) and changes in biomarkers concentration of the MultiVeg study participants from baseline to 4 months (t2-t0)

Nutrient biomarker [unit]		CON (*n* = 37)	INT (*n* = 35)	*p*-values ^a^	*p*-values (baseline-adjusted)^b^	*p*-values (adjusted for baseline, age, and sex)^c^	*p*-values (multi-adjusted)^d^
Vitamin A [mg/L]	t0^e^	0.47 (0.20)	0.48 (0.14)	1			
	t2^e^	0.68 (0.23)	0.69 (0.20)	1			
	t2-t0^e^	0.17 (0.15)	0.20 (0.17)	1	1	1	1
RBP [mg/dL]	t0^e^	1.90 (0.7)	2.00 (0.5)	1			
	t2^e^	1.72 (0.53)	1.99 (0.53)	1			
	t2-t0^e^	− 0.29 (0.30)	− 0.11 (0.29)	1	1	1	0.940
TTR [g/L]	t0^f^	0.26 ± 0.06	0.25 ± 0.04	1			
	t2^f^	0.23 ± 0.05	0.23 ± 0.05	1			
	t2-t0^f^	− 0.03 ± 0.03	− 0.02 ± 0.05	1	1	1	1
Beta-carotene [µg/L]	t0^e^	331.0 (427.0)	409.0 (347.0)	1			
	t2^e^	416.0 (257.0)	369.0 (307.0)	1			
	t2-t0^e^	23.0 (285.0)	1.0 (219.0)	1	1	1	1
FAD [µg/L]	t0^e^	246.0 (43.0)	232.0 (31.0)	1			
	t2^f^	194.1 ± 24.6	212.8 ± 24.1	0.204			
	t2-t0^e^	− 46.0 (24.0)	− 18.0 (32.0)	**0.008**	**< 0.001**	**< 0.001**	**< 0.001**
Vitamin B_12_ [pg/mL]	t0^e^	369.0 (266.0)	330.0 (204)	1			
	t2^e^	293.0 (187.0)	376.0 (159.0)	1			
	t2-t0^e^	− 73.0 (83.5)	38.0 (158.0)	**0.029**	**0.011**	**0.016**	**0.013**
HoloTC [pmol/L]	t0^e^	58.0 (37.0)	50.0 (25.0)	1			
	t2^e^	72.0 (28.0)	83.0 (20.0)	1			
	t2-t0^e^	11.0 (16.0)	34.0 (24.0)	**0.007**	**< 0.001**	**< 0.001**	**< 0.001**
MMA [µg/L]	t0^e^	18.0 (12.0)	20.0 (7.0)	1			
	t2^e^	14.0 (6.0)	11.0 (5.0)	1			
	t2-t0^e^	− 6.0 (7.0)	-8.0 (5.0)	1	**0.048**	0.051	0.069
Hcy [µmol/L]	t0^e^	10.0 (4.0)	10.1 (5.0)	1			
	t2^e^	10.4 (4.3)	9.2 (4.8)	1			
	t2-t0^e^	1.3 (2.5)	− 0.3 (4.5)	0.5	0.202	0.103	0.104
cB_12_	t0^f^	0.29 ± 0.58	0.17 ± 0.52	1			
	t2^f^	0.34 ± 0.53	0.68 ± 0.45	0.495			
	t2-t0^f^	0.06 ± 0.25	0.51 ± 0.44	**< 0.001**	**< 0.001**	**< 0.001**	**< 0.001**
Choline [µg/L]	t0^e^	746.0 (169.0)	751.0 (182.0)	1			
	t2^e^	901.0 (238.0)	940.0 (242.0)	1			
	t2-t0^f^	126.6 ± 175.7	224.5 ± 209.8	1	1	1	NA^g^
Folate [µg/L]	t0^e^	389.0 (82.0)	376.0 (117.0)	1			
	t2^e^	332.0 (79.0)	342.0 (123.0)	1			
	t2-t0^e^	− 61.0 (55.0)	-45.0 (63.0)	1	1	1	1
OC [ng/mL]	t0^f^	29.6 ± 9.8	32.0 ± 9.9	1			
	t2^f^	28.2 ± 7.5	27.4 ± 8.6	1			
	t2-t0^f^	− 1.4 ± 5.5	− 4.6 ± 6.3	1	1	1	1
cOC [ng/mL]	t0^e^	11.4 (6.6)	12.5 (5.1)	1			
	t2^e^	11.9 (6.2)	11.7 (6.7)	1			
	t2-t0^e^	− 0.19 (3.2)	0.10 (3.2)	1	1	1	1
ucOC [ng/mL]	t0^e^	10.6 (11.4)	11.2 (8.4)	1			
	t2^e^	8.5 (6.1)	7.6 (6.5)	1			
	t2-t0^f^	− 1.6 ± 5.4	− 3.4 ± 3.9	1	1	1	1

RBP: retinol-binding protein; TTR: transthyretin; FAD: flavin adenine dinucleotide; HoloTC: holotranscobalamin; MMA: methylmalonic acid; Hcy: homocysteine; cB_12_: combined indicator; NA: not available; OC: total osteocalcin; cOC: carboxylated osteocalcin; ucOC: undercarboxylated osteocalcin

^a^ Independent t-test (normally distributed continuous variables) or Mann-Whitney U test (non-normally distributed continuous variables). ^b^ ANCOVA, adjusted for baseline blood values. ^c^ ANCOVA, adjusted for baseline blood values, age, and sex. ^d^ ANCOVA, adjusted for baseline blood values, age, sex, and mean dietary nutrient intake from t0 and t2 (vitamin A, RBP, TTR, and beta-carotene were adjusted for vitamin A intake [as retinol equivalent], FAD for vitamin B_2_ intake, vitamin B_12_, holoTC, MMA, Hcy and cB_12_ for vitamin B_12_ intake, folate for folate intake [as dietary folate equivalent] and OC, cOC, and ucOC for the intake of vitamin K, vitamin D, and calcium [the last two nutrients are covered in detail in other articles])

All *p*-values are adjusted for multiple testing by Bonferroni-Holm correction. Bold values indicate statistically significant results (p < 0.05)

^e^ Median (IQR). ^f^ Mean ± SD. ^g^ Choline cannot be adjusted for choline intake, as there are no food composition data available for choline in the German Nutrient Database (Bundeslebensmittelschlüssel BLS 3.02)

## Discussion

In the present study, the multinutrient supplement improved the vitamin B_12_ status of healthy vegans. Although it did not increase it, it also had an influence on vitamin B_2_ status (FAD) as the decline in FAD levels was less pronounced in the INT compared to the CON. Despite these observations, the supplement did not significantly affect the blood levels of vitamin A, RBP, TTR, beta-carotene, choline, folate, OC, cOC, and ucOC.

After 4 months of multinutrient supplementation, there were no significant changes in the relevant parameters of vitamin A status between the groups. Both groups experienced a non-significant increase in serum retinol, but a decrease in RBP. However, TTR changes were similar in both groups. Overall, RBP and TTR parameters were consistently low before and after the intervention in comparison to the reference values (Tables 4 and 7), suggesting an overall poor vitamin A status.

Nevertheless, beta-carotene increased more, but non-significantly in the CON than in the INT. This can be explained by a higher dietary intake of beta-carotene: At all three time points median dietary nutrient intakes of RE (mostly beta-carotene [19]) were slightly higher in the CON than in the INT. Administering a multinutrient supplement with 743 µg of vitamin A (measured, declared 500 µg) may slightly preserve the vitamin A status, but it might not significantly improve vitamin A status within 4 months.

For vitamin B_2_, there are several plant sources, e.g., nuts, mushrooms, legumes, yeast flakes, or fortified plant-based dairy alternatives [2]. Despite conflicting study results, vitamin B_2_ may be considered a critical nutrient in vegan diets. In a cross-sectional study from Switzerland, there was no significant difference in vitamin B_2_ plasma levels between vegans, lacto-ovo-vegetarians (LOV), and omnivores [15]. In contrast, a cross-sectional study from Germany demonstrated lower plasma vitamin B_2_ levels among vegans in comparison to omnivores. However, the median concentration in both groups exceeded the established reference value [40]. Conversely, in a cross-sectional study from Austria from 2006, approximately 30% of vegan participants had vitamin B_2_ levels below the reference value. This was in contrast to around 10% observed among both LOV and omnivorous participants [41]. Comparably, in the present study, the CON showed a decrease in FAD levels, falling just below the reference range (199–382 µg/L). Moreover, a decline in vitamin B_2_ status from t0 to t2 was observed in both the INT and CON. However, the reduction in status was significantly less pronounced in the INT, suggesting that the multinutrient supplement may have attenuated the decrease in vitamin B_2_ status compared to placebo. One explanation might be a lower intake as both groups reduced their vitamin B_2_ intake during the study from t0 to t1. However, it rebounded in both groups at t2. Changes (possibly related to infection, genetics or exercise) in FAD levels are another hypothetical explanation, but these have not been well studied [42]. Furthermore, the supplement’s dosage (1.03 mg vitamin B_2_) might not be sufficient to maintain the vitamin B_2_ status in the long term, even though the INT was still within the reference range due to a lower decrease compared to the CON. Thus, monitoring over an extended period is recommended.

For vitamin B_12_, the intergroup difference of the serum vitamin B_12_ concentration, a less specific and sensitive marker of vitamin B_12_ status [33], was significant (INT: +38 pg/mL, CON − 73 pg/mL). Similarly, holoTC, an early indicator of availability of vitamin B_12_ to body cells, increased significantly more in the INT compared to the CON (+ 34.0 pmol/L, CON + 11.0 pmol/L). Although the CON did not receive vitamin B_12_, the rise in holoTC levels in the CON may be due to various factors. The dietary intake of vitamin B_12_ on average was 9–12%DACH (CON) and 2–10%DACH (INT) (Table 6), thus, might not explain a rise in holoTC. However, it also cannot be ruled out that vitamin B_12_-enriched foods were consumed beyond those recorded in the dietary protocols, leading to slight increases in holoTC levels. Both groups maintained holoTC levels within the reference range throughout the study, indicating sufficient vitamin B_12_ stores. Serum vitamin B_12_ levels may decrease before holoTC changes due to enterohepatic recycling maintaining holoTC concentrations or may even slightly increase them until stores are considerably depleted [22, 43, 44]. Additionally, a negative vitamin B_12_ balance in the CON may affect holoTC levels differently than an outright vitamin B_12_ deficiency. An abrupt cessation of dietary vitamin B_12_ intake can occur when transitioning from an omnivorous to an unsupplemented vegan diet. In the context of the current study, some participants may have stopped taking their usual supplements before the recruitment phase in order to fulfil the inclusion criteria and participate in the study. This scenario is possible, given that data suggest 82–92% of vegans in Germany consume vitamin B_12_ supplements or vitamin-B_12_-fortified foods [40, 45]. Moreover, MMA levels decreased (only significant in the baseline-adjusted model) and Hcy decreased more in the INT compared to the CON (but not statistically significant) and over the 4-month period. Both folate and vitamin B_12_ are crucial for the regulation of homocysteine levels [46]. Two outliers in the INT exhibited notably elevated Hcy levels at t2 and had one of the lowest serum folate levels of all participants. Overall, as the multinutrient supplement contained no folate, no significant differences were found for the vitamin between the groups at either time point (t0 and t2) and in terms of changes (t0 to t2), as expected. The median Hcy levels in both groups were within the reference range before and after the intervention. For both groups, values for cB_12_ remained within the reference range indicating vitamin B_12_ adequacy (-0.5 to 1.5 [23]), with the INT demonstrating a statistically significant improvement over time in comparison to the CON. Vitamin B_12_ is regarded as the most critical nutrient in vegan diets [47], so the observed change in vitamin B_12_ status with vitamin B_12_ supplementation compared to placebo was to be expected. It should be noted that a small percentage of the participants reported taking vitamin B_12_ (CON 13.5%, INT 14.3%) within the study’s allowed limits (Recruitment Section, Supplementary Table S2). However, this intake was not confirmed in the 3-day weighed dietary records, possibly because the subjects consumed the supplement irregularly or just not during the recorded days.

In vegan diets, vitamin K is not considered a critical nutrient [2] as there are various plant sources of this vitamin, e.g., dark green leafy vegetables or soya milk [48]. The results of the present study demonstrate that the OC levels in the INT declined more compared to the CON. However, this difference was not significant, especially when adjusting for both baseline blood values, intake of vitamin K, vitamin D, and calcium, age, and sex. Because of their roles in bone health [49–51], vitamin D and calcium were considered as confounders and future articles will focus on both nutrients. Nevertheless, vitamin D supplementation had a significant effect on 25-OH-D3 compared with placebo (data not shown). Besides, no significant difference was observed for cOC or cOC changes over time between the INT and CON. In the INT, ucOC levels decreased more than in the CON, but not significantly. However, these findings are consistent with the observation that vitamin K_2_ supplementation decreases serum levels of ucOC, which indicates an increase in cOC [29]. Concerning the within-group changes of OC, cOC, and ucOC levels, no significant differences were observed between t0 and t2. Both groups were within the laboratory’s reference range for OC levels, both at t0 and t2, while there are none for cOC and ucOC. This suggests that the influence of the supplement was not clearly demonstrable or may have been influenced by other factors. For instance, there was no need for vitamin K_2_ supplementation as the dietary vitamin K intake was already sufficient.

Choline is currently discussed as a potentially critical nutrient in vegan diets [16] because animal foods such as eggs and meat are rich dietary sources [17]. The human body can synthesize choline, but there are various genetic polymorphisms that can limit its endogenous synthesis capability [52]. The increase in serum choline levels in the INT was greater, but not significantly. One reason for the increase in serum choline levels in the CON could be a higher dietary intake which we were unfortunately unable to assess. It should be noted that some participants in both groups reported occasional consumption of animal products in the recruitment questionnaire (Supplementary Table S2), particularly eggs (31.4% CON, 20.0% INT < 1/month; 2.7% CON, 17.1% INT 1–3/month), which may explain the observed choline levels. However, this intake was not confirmed in the 3-day weighed dietary records. Another reason might be a greater endogenous synthesis capability in the CON. In addition, compliance was only moderate in both groups with 54–60% following the study protocol. This could have caused the lack of significant differences between the groups. A sufficient folate status is linked to higher plasma choline levels [53]. As there was no significant difference in the folate status, this cannot explain the lack of difference. Furthermore, both groups were within the reference range for plasma choline before and after the intervention, underlining that neither plasma choline levels nor other parameters are reliable biomarkers for assessing choline status [27, 54]. Alternatively, this may suggest that a vegan diet provides sufficient dietary choline to meet dietary requirements. However, further explanation or investigation is needed to fully understand this finding.

### Strengths and limitations

The MultiVeg study makes an important contribution to the field of vegan nutrition because, to our knowledge, it is the first RCT testing a multinutrient supplement in healthy vegans. This is important as there is a broad variety of multinutrient supplements for vegans on the market. Another strength is the similarity of both study groups with no significant differences in baseline characteristics. A further strength is the detailed dietary assessment.

A limitation lies in the recruitment process. Participants were mainly recruited through posts on social media (convenience sample) and via a YouTube live event which included a speaker of the funding company which also markets the multinutrient supplements. This should be kept in mind when interpreting the results as it appears to limit the generalizability of the results.

Furthermore, most participants appear to have an adequate nutrient status at baseline, with over 75% of individuals in each group within or above the reference ranges for most nutrients (below or within the recommended value[s] for Hcy and MMA). This pre-existing adequate nutrient status likely contributed to the observation that, after the intervention, still over 75% of participants in both groups remained within the reference ranges. However, in the INT, fewer participants fell outside these ranges, with many shifting further up within the reference intervals, while in the CON, more participants fell outside the reference ranges or shifted downward in regards of their nutrient status, although many remained within the acceptable limits (Supplementary Table S1).

Another limitation is the study protocol which required a daily intake of three capsules and a powder, which may have had a detrimental impact on participants’ compliance and adherence to the intervention regimen. This is evidenced by the moderate compliance with the choline supplement. This moderate adherence highlights the potential challenges associated with following a vegan diet, which may require multiple supplements to meet nutrient needs, and may have influenced the study’s findings on serum choline levels. Given that it is not yet fully established that choline is a critical nutrient for vegans, it is possible that the inclusion of this nutrient in the intervention could be reconsidered. Without the choline powder, participants would have only needed to take two supplements, which could have potentially improved compliance. In contrast, compliance in capsule form was high (> 94% of participants in both groups taking it 6–7 times a week), highlighting that the capsule format may be more feasible for regular use than a powdered supplement. Also, compliance was only assessed at the end of the study and not at the midpoint, which may limit our ability to fully evaluate adherence throughout the entire intervention period.

A further limitation of our study could be the 16-week duration, which may not have been long enough to observe the potential long-term impact of the supplement on every analysed nutrient. However, for key parameters like vitamin B_12_ and vitamin A, previous studies have demonstrated that significant changes can also be observed even within 12–16 weeks [36, 55–57], suggesting that the study duration was sufficient to assess the primary outcomes.

Furthermore, the inclusion of participants without diagnosed deficiencies may have constrained the capacity to evaluate the supplements’ efficacy in addressing deficiencies. To address these limitations, we mainly included interested individuals taking no or few supplements prior to the study. Nevertheless, the participants mostly had a normal baseline status. Therefore, it was anticipated that there might be little to no change in many nutrient levels, which aligns with the findings for several nutrients for which no significant impact was observed.

In our study, 3-day weighed dietary records were utilised, with participants using their own kitchen scales. This could introduce measurement bias due to potential inaccuracies in reported portion sizes, influencing the reliability of reported dietary nutrient intake data. Furthermore, currently there is no database that includes choline concentrations in foods in Germany. Therefore, no statements could be made regarding dietary choline intake in this study. Additionally, a considerable number of processed vegan products consumed by the participants are not yet included in the German Nutrient Database (BLS). Consequently, we were obliged to simulate these foods based on their declared ingredients and provided information regarding their energy and macronutrient content. This method permitted the estimation of micronutrient content, which is often absent from product labels. However, this approach is susceptible to inaccuracies. Nevertheless, it represents the most viable option within the context of dietary surveys [58].

## Conclusions

In conclusion, the daily intake of the investigated multinutrient supplement with a dosage of 82 µg vitamin B_12_ appears to constitute a reliable approach to increase vitamin B_12_ status among healthy vegans. As there are currently no standardised reference values for sufficient vitamin B_12_ intake via supplements, these results might help to establish vitamin B_12_ intake recommendations for vegans. In contrast, no clear positive impact of the multinutrient supplement on nutrient status could be demonstrated for all other examined vitamins and choline, leading to the assumption that the participants were already adequately supplied with these nutrients. The overall poor vitamin A status of the participants supports the suggestions that vitamin A should be included in the group of potentially critical nutrients in a vegan diet and the dose of a vitamin A supplement should be higher than in the present supplement (i.e., >  750 µg per day). The lack of improvement in vitamin A and B_2_ status might be due to interactions with other nutrients that may decrease their bioavailability in a multinutrient supplement and/or insufficient amounts in the supplement. Although our data show that well-nourished vegans do not benefit from additional multivitamin supplementation, it may still be worth considering as a prophylactic measure. However, the feasibility of administering three different supplements per day should be reconsidered, as compliance may be a challenge in practical settings.

## Supplementary Information

Below is the link to the electronic supplementary material.

Supplementary Material 1

Supplementary Material 2

## Data Availability

The dataset is available upon reasonable request from the corresponding author.

## References

[1] Dittmann A, Werner L, Storcksdieck genannt Bonsmann S, Hoffmann I (2023) How high is the share of vegetarian and vegan diets in germany? An exploration of the study situation. Ernähr Umsch 70(7):80–93. 10.4455/eu.2023.012

[2] Richter M, Boeing H, Grünewald-Funk D et al (2016) Vegan diet position of the German nutrition society (DGE). Ernähr Umsch 63(04):92–102. 10.4455/eu.2016.021. Erratum in: 63(05):M262

[3] Arterburn LM, Hall EB, Oken H (2006) Distribution, interconversion, and dose response of n – 3 fatty acids in humans. Am J Clin Nutr 83:1467S-1476S. 10.1093/ajcn/83.6.1467S 10.1093/ajcn/83.6.1467S 16841856

[4] Hovinen T, Korkalo L, Freese R et al (2021) Vegan diet in young children remodels metabolism and challenges the statuses of essential nutrients. EMBO Mol Med 13(2):1–12. 10.15252/emmm.202013492 10.15252/emmm.202013492 PMC7863396 33471422

[5] IOM (2001) Dietary reference intakes for vitamin A, vitamin K, arsenic, boron, chromium, copper, iodine, iron, manganese, molybdenum, nickel, silicon, vanadium, and zinc. National Academy, Washington, D.C 25057538

[6] Lietz G, Lange J, Rimbach G (2010) Molecular and dietary regulation of β,β-carotene 15,15′-monooxygenase 1 (BCMO1). Arch Biochem Biophys 502:8–16. 10.1016/j.abb.2010.06.032 20599666 10.1016/j.abb.2010.06.032

[7] Hickenbottom SJ, Follett JR, Lin Y et al (2002) Variability in conversion of β-carotene to vitamin A in men as measured by using a double-tracer study design. Am J Clin Nutr 75:900–907. 10.1093/ajcn/75.5.900 11976165 10.1093/ajcn/75.5.900

[8] Leung WC, Hessel S, Méplan C et al (2009) Two common single nucleotide polymorphisms in the gene encoding β-carotene 15,15′‐monoxygenase alter β‐carotene metabolism in female volunteers. FASEB J 23:1041–1053. 10.1096/fj.08-121962 19103647 10.1096/fj.08-121962

[9] Lindqvist A, Sharvill J, Sharvill DE, Andersson S (2007) Loss-of-Function mutation in carotenoid 15,15´-Monooxygenase identified in a patient with hypercarotenemia and hypovitaminosis A. J Nutr 137:2346–2350. 10.1093/jn/137.11.2346 17951468 10.1093/jn/137.11.2346

[10] Penniston KL, Tanumihardjo SA (2006) The acute and chronic toxic effects of vitamin A. Am J Clin Nutr 83:191–201. 10.1093/ajcn/83.2.191 16469975 10.1093/ajcn/83.2.191

[11] EFSA (2017) Dietary reference values for nutrients summary report. EFSA Support Publ 2017(e15121):92. 10.2903/sp.efsa.2017.e15121

[12] Koeder C, Perez-Cueto FJA (2022) Vegan nutrition: a preliminary guide for health professionals. Crit Rev Food Sci Nutr 1–38. 10.1080/10408398.2022.2107997 10.1080/10408398.2022.2107997 35959711

[13] Olsen A, Halkjær J, Van Gils CH et al (2009) Dietary intake of the water-soluble vitamins B1, B2, B6, B12 and C in 10 countries in the European prospective investigation into cancer and nutrition. Eur J Clin Nutr 63:S122–S149. 10.1038/ejcn.2009.78 19888270 10.1038/ejcn.2009.78

[14] Sobiecki JG, Appleby PN, Bradbury KE, Key TJ (2016) High compliance with dietary recommendations in a cohort of meat eaters, fish eaters, vegetarians, and vegans: results from the European prospective investigation into cancer and Nutrition–Oxford study. Nutr Res 36:464–477. 10.1016/j.nutres.2015.12.016 27101764 10.1016/j.nutres.2015.12.016 PMC4844163

[15] Schüpbach R, Wegmüller R, Berguerand C et al (2017) Micronutrient status and intake in omnivores, vegetarians and vegans in Switzerland. Eur J Nutr 56:283–293. 10.1007/s00394-015-1079-7 26502280 10.1007/s00394-015-1079-7

[16] Roeren M, Kordowski A, Sina C, Smollich M (2022) Inadequate choline intake in pregnant women in Germany. Nutrients 14:4862. 10.3390/nu14224862 36432547 10.3390/nu14224862 PMC9696170

[17] Vennemann FBC, Ioannidou S, Valsta LM et al (2015) Dietary intake and food sources of choline in European populations. Br J Nutr 114:2046–2055. 10.1017/S0007114515003700 26423357 10.1017/S0007114515003700

[18] Kurtha A, Ellis F (1971) Investigation into the causation of the electroencephalogram abnormality in vegans. Plant Foods Hum Nutr 53–59

[19] Tang G (2010) Bioconversion of dietary provitamin A carotenoids to vitamin A in humans. Am J Clin Nutr 91. 10.3945/ajcn.2010.28674G. :1468S-1473S 10.3945/ajcn.2010.28674G PMC2854912 20200262

[20] Ronne H, Ocklind C, Wiman K et al (1983) Ligand-dependent regulation of intracellular protein transport: effect of vitamin a on the secretion of the retinol-binding protein. JCB 96:907–910. 10.1083/jcb.96.3.907 6682115 10.1083/jcb.96.3.907 PMC2112407

[21] Engle-Stone R, Haskell MJ, Ndjebayi AO et al (2011) Plasma retinol-Binding protein predicts plasma retinol concentration in both infected and uninfected Cameroonian women and children. J Nutr 141:2233–2241. 10.3945/jn.111.145805 22049292 10.3945/jn.111.145805

[22] Carmel R (2011) Biomarkers of cobalamin (vitamin B-12) status in the epidemiologic setting: a critical overview of context, applications, and performance characteristics of cobalamin, methylmalonic acid, and holotranscobalamin II. Am J Clin Nutr 94:348S–358S. 10.3945/ajcn.111.013441 21593511 10.3945/ajcn.111.013441 PMC3174853

[23] Fedosov SN, Brito A, Miller JW et al (2015) Combined indicator of vitamin B12 status: modification for missing biomarkers and folate status and recommendations for revised cut-points. Clin Chem Lab Med (CCLM) 53. 10.1515/cclm-2014-0818 10.1515/cclm-2014-0818 25720072

[24] Speek AJ, Van Schaik F, Schrijver J, Schreurs WHP (1982) Determination of the B2 vitamer flavin—adenine dinucleotide in whole blood by high-performance liquid chromatography with fluorometric detection. J Chromatogr B Biomed 228:311–316. 10.1016/S0378-4347(00)80446-6 10.1016/s0378-4347(00)80446-6 7076754

[25] Arppe R, Mattsson L, Korpi K et al (2015) Homogeneous assay for whole blood folate using photon upconversion. Anal Chem 87:1782–1788. 10.1021/ac503691m 25548870 10.1021/ac503691m

[26] Dueker SR, Lin Y, Jones AD et al (2000) Determination of blood folate using acid extraction and internally standardized gas Chromatography–Mass spectrometry detection. Anal Biochem 283:266–275. 10.1006/abio.2000.4660 10906248 10.1006/abio.2000.4660

[27] EFSA (2016) Dietary reference values for choline. EFS2 14. 10.2903/j.efsa.2016.4484

[28] Zeisel SH (2000) Choline: an essential nutrient for humans. Nutrition 16:669–671. 10.1016/S0899-9007(00)00349-X 10906592 10.1016/s0899-9007(00)00349-x

[29] Gundberg CM, Lian JB, Booth SL (2012) Vitamin K-Dependent carboxylation of osteocalcin: friend or foe? Adv Nutr 3:149–157. 10.3945/an.112.001834 22516722 10.3945/an.112.001834 PMC3648715

[30] DGE ÖGE (2024) Reference values for nutrient intake, 2nd edn, 8th updated edition. Neuer Umschau Buchverlag, Bonn

[31] WHO (2012) Serum retinol concentrations for determining the prevalence of vitamin A deficiency in populations. Vitamin and mineral nutrition information system, Geneva

[32] Dati F, Schumann G, Thomas L et al (1996) Consensus of a group of professional societies and diagnostic companies on guidelines for interim reference ranges for 14 proteins in serum based on the standardization against the IFCC/BCR/CAP reference material (CRM 470). International federation of clinical Chemistry. Community bureau of reference of the commission of the European Communities. College of American pathologists. Eur J Clin Chem Clin Biochem 34:517–520 8831057

[33] Herrmann W, Obeid R (2008) Causes and early diagnosis of vitamin B12 deficiency. Dtsch Arztebl Int. 10.3238/arztebl.2008.0680 10.3238/arztebl.2008.0680 PMC2696961 19623286

[34] Kirsch SH, Herrmann W, Rabagny Y, Obeid R (2010) Quantification of acetylcholine, choline, betaine, and dimethylglycine in human plasma and urine using stable-isotope Dilution ultra performance liquid chromatography–tandem mass spectrometry. J Chromatogr B 878:3338–3344. 10.1016/j.jchromb.2010.10.016 10.1016/j.jchromb.2010.10.016 21074502

[35] WHO (2012) Serum and red blood cell folate concentrations for assessing folate status in populations. Vitamin and mineral nutrition information system, Geneva

[36] Siebert A-K, Obeid R, Weder S et al (2017) Vitamin B-12–fortified toothpaste improves vitamin status in vegans: a 12-wk randomized placebo-controlled study. Am J Clin Nutr 105:618–625. 10.3945/ajcn.116.141978 28052884 10.3945/ajcn.116.141978

[37] Twisk J, Bosman L, Hoekstra T et al (2018) Different ways to estimate treatment effects in randomised controlled trials. Contemp Clin Trials Commun 10:80–85. 10.1016/j.conctc.2018.03.008 29696162 10.1016/j.conctc.2018.03.008 PMC5898524

[38] Victor A, Elsäßer A, Hommel G, Blettner M (2010) Judging a plethora of* p*-Values. Dtsch Arztebl Int 107(4):50–56. 10.3238/arztebl.2010.0050 20165700 10.3238/arztebl.2009.0050 PMC2822959

[39] Hubbard GP, Elia M, Holdoway A, Stratton RJ (2012) A systematic review of compliance to oral nutritional supplements. Clin Nutr 31:293–312. 10.1016/j.clnu.2011.11.020 22257636 10.1016/j.clnu.2011.11.020

[40] Weikert C, Trefflich I, Menzel J et al (2020) Vitamin and mineral status in a vegan diet. Dtsch Arztebl Int 117:575–582. 10.3238/arztebl.2020.0575 33161940 10.3238/arztebl.2020.0575 PMC7779846

[41] Majchrzak D, Singer I, Männer M et al (2006) B-Vitamin status and concentrations of homocysteine in Austrian Omnivores, vegetarians and vegans. Ann Nutr Metab 50:485–491. 10.1159/000095828 16988496 10.1159/000095828

[42] Mosegaard S, Dipace G, Bross P et al (2020) Riboflavin Deficiency—Implications for general human health and inborn errors of metabolism. Int J Mol Sci 21:3847. 10.3390/ijms21113847 32481712 10.3390/ijms21113847 PMC7312377

[43] Golding PH (2016) Holotranscobalamin (HoloTC, Active-B12) and herbert’s model for the development of vitamin B12 deficiency: a review and alternative hypothesis. SpringerPlus 5:668. 10.1186/s40064-016-2252-z 27350907 10.1186/s40064-016-2252-z PMC4899389

[44] Lederer A-K, Hannibal L, Hettich M et al (2019) Vitamin B12 status upon Short-Term intervention with a vegan diet—a randomized controlled trial in healthy participants. Nutrients 11:2815. 10.3390/nu11112815 31752105 10.3390/nu11112815 PMC6893687

[45] Vollmer I, Keller M, Kroke A (2018) Vegan diet: utilization of dietary supplements and fortified foods. An internet-based survey. Ernähr Umsch 65(9):144–153. 10.4455/eu.2018.030

[46] Shane B, Stokstad ELR (1985) Vitamin B_12_-Folate interrelationships. Annu Rev Nutr 5:115–141. 10.1146/annurev.nu.05.070185.000555 3927946 10.1146/annurev.nu.05.070185.000555

[47] Allen LH, Miller JW, De Groot L et al (2018) Biomarkers of nutrition for development (BOND): vitamin B-12 review. J Nutr 148. 10.1093/jn/nxy201. :1995S–2027S 10.1093/jn/nxy201 PMC6297555 30500928

[48] Hess JM (2022) Modeling dairy-free vegetarian and vegan USDA food patterns for Nonpregnant, nonlactating adults. J Nutr 152:2097–2108. 10.1093/jn/nxac100 35485767 10.1093/jn/nxac100

[49] Suksomboon N, Poolsup N, Darli Ko Ko H (2017) Effect of vitamin K supplementation on insulin sensitivity: a meta-analysis. DMSO 10:169–177. 10.2147/DMSO.S137571 10.2147/DMSO.S137571 PMC5422317 28496349

[50] Lin X, Brennan-Speranza TC, Levinger I, Yeap BB (2018) Undercarboxylated osteocalcin: experimental and human evidence for a role in glucose homeostasis and muscle regulation of insulin sensitivity. Nutrients 10:847. 10.3390/nu10070847 29966260 10.3390/nu10070847 PMC6073619

[51] Shea MK, Booth SL, Massaro JM et al (2007) Vitamin K and vitamin D status: associations with inflammatory markers in the Framingham offspring study. Am J Epidemiol 167:313–320. 10.1093/aje/kwm306 18006902 10.1093/aje/kwm306 PMC3151653

[52] Zeisel SH (2007) Gene response elements, genetic polymorphisms and epigenetics influence the human dietary requirement for choline. IUBMB Life 59:380–387. 10.1080/15216540701468954 17613168 10.1080/15216540701468954 PMC2430110

[53] Konstantinova SV, Tell GS, Vollset SE et al (2008) Divergent associations of plasma choline and betaine with components of metabolic syndrome in middle age and elderly men and women. J Nutr 138:914–920. 10.1093/jn/138.5.914 18424601 10.1093/jn/138.5.914

[54] IOM (1998) Dietary reference intakes for thiamin, riboflavin, niacin, vitamin B_6_, folate, vitamin B_12_, pantothenic acid, biotin, and choline. National Academy, Washington, D.C 23193625

[55] Li Z, Fan P, Deng G et al (2012) Effects of vitamin A supplementation on nutritional status of iron in healthy adults. Wei Sheng Yan Jiu 41:419–423 23050440

[56] Del Bo’ C, Riso P, Gardana C et al (2019) Effect of two different Sublingual dosages of vitamin B12 on cobalamin nutritional status in vegans and vegetarians with a marginal deficiency: a randomized controlled trial. Clin Nutr 38:575–583. 10.1016/j.clnu.2018.02.008 29499976 10.1016/j.clnu.2018.02.008

[57] Ding Y, Hu P, Yang Y et al (2021) Impact of maternal daily oral Low-Dose vitamin A supplementation on the Mother-Infant pair: A randomised Placebo-Controlled trial in China. Nutrients 13:2370. 10.3390/nu13072370 34371880 10.3390/nu13072370 PMC8308679

[58] Sichert-Hellert W, Kersting M, Chahda C et al (2007) German food composition database for dietary evaluations in children and adolescents. J Food Compost Anal 20:63–70. 10.1016/j.jfca.2006.05.004

