# Supplementary material for: Assessment of vitamin A, vitamin B2, vitamin B12, vitamin K, folate, and choline status following 4 months of multinutrient supplementation in healthy vegans: a randomised, double-blind, placebo-controlled trial
Source: Eur J Nutr. 2025 Dec 19;65(1):9. doi: 10.1007/s00394-025-03814-7 (PMC12717231; doi:10.1007/s00394-025-03814-7)
Supplement: Supplementary file 1 — Supplementary Material 1 [file 394_2025_3814_MOESM1_ESM.docx]

**Assessment of Vitamin A, Vitamin B2, Vitamin B12, Vitamin K, Folate, and Choline Status Following 4 Months of Multinutrient Supplementation in Healthy Vegans: A Randomised, Double-Blind, Placebo-Controlled Trial**

Tim Zerback^1^, Christian Koeder^2,1^*, Stine Weder^1^, Andreas Sputtek^3^, Gunter P. Eckert^4^, Markus Keller^1^

^1^ Research Institute For Plant-Based Nutrition, 35444 Biebertal, Germany, ORCID: TZ 0009-0003-0743-5736, SW 0000-0001-5360-1940, MK 0000-0001-5789-6280

^2^ Corresponding author: Institute for Prevention and Cancer Epidemiology (IPE), Medical Center - University of Freiburg, Faculty of Medicine, University of Freiburg, Elsässerstr. 2, 79110 Freiburg im Breisgau, Germany, E-Mail: [christian.koeder@uniklinik-freiburg.de](mailto:christian.koeder@uniklinik-freiburg.de), ORCID: 0000-0002-3761-4220

^3^ MVZ Medical Laboratory Bremen GmbH, 28359 Bremen, Germany

^4^ Laboratory for Nutrition in Prevention and Therapy, Biomedical Research Center Seltersberg (BFS), Institute of Nutritional Sciences, Justus-Liebig-University of Giessen, Germany, ORCID: 0000-0002-8002-9983

# Summary

Table S1 presents the percentage of participants with circulating biomarkers outside of the reference ranges. Table S2 shows the frequency of animal product and supplement consumption within the study's allowed limits among participants. Table S3 presents the average nutrient biomarkers concentration at baseline (t0) and after 4 months (t2) and intragroup differences in biomarkers concentration of the MultiVeg study participants from baseline to 4 months (t2-t0).

Supplementary Materials

**Supplemental Table S1** Absolute number and percentage of participants with circulating biomarkers outside of the reference ranges

| **Nutrient biomarker [unit]** |  | **Number (%) <reference range** | | **Number (%) >reference range** | |
| --- | --- | --- | --- | --- | --- |
|  |  | **CON (n=37)** | **INT (n=35)** | **CON (n=37)** | **INT (n=35)** |
| Vitamin A [mg/L] | t0 | 0 (0) | 0 (0) | 0 (0) | 0 (0) |
|  | t2 | 0 (0) | 0 (0) | 0 (0) | 0 (0) |
| RBP [mg/dL] | t0 | 22 (60) | 19 (54) | 0 (0) | 0 (0) |
|  | t2 | 28 (76) | 21 (60) | 0 (0) | 0 (0) |
| TTR [g/L] | t0 | 5 (14) | 3 (9) | 1 (3) | 0 (0) |
|  | t2 | 11 (30) | 8 (23) | 0 (0) | 0 (0) |
| Beta-carotene [µg/L] | t0 | 2 (5) | 3 (9) | 1 (3) | 0 (0) |
|  | t2 | 2 (5) | 3 (9) | 0 (0) | 0 (0) |
| FAD [µg/L] | t0 | 5 (14) | 2 (6) | 0 (0) | 0 (0) |
|  | t2 | 19 (51) | 8 (23) | 0 (0) | 0 (0) |
| Vitamin B_12_ [pg/mL] | t0 | 3 (8) | 4 (11) | 1 (3) | 1 (3) |
|  | t2 | 6 (16) | 5 (14) | 0 (0) | 1 (3) |
| HoloTC [pmol/L] | t0 | 0 (0) | 1 (3) | 2 (5) | 1 (3) |
|  | t2 | 0 (0) | 0 (0) | 0 (0) | 0 (0) |
| MMA [µg/L] | t0 | 0 (0) | 0 (0) | 6 (16) | 3 (9) |
|  | t2 | 4 (11) | 4 (11) | 4 (11) | 0 (0) |
| Hcy [µmol/L] | t0 | NA^a^ | | 6 (16) | 8 (23) |
|  | t2 |  |  | 7 (19) | 7 (20) |
| cB_12_^b^ | t0 | 4 (11) | 5 (14) | 1 (3) | 0 (0) |
|  | t2 | 3 (8) | 0 (0) | 0 (0) | 0 (0) |
| Choline [µg/L] | t0 | 16 (43) | 16 (46) | 1 (3) | 0 (0) |
|  | t2 | 8 (22) | 3 (9) | 2 (5) | 1 (3) |
| Folate [µg/L] | t0 | 0 (0) | 0 (0) | 3 (8) | 4 (11) |
|  | t2 | 0 (0) | 0 (0) | 2 (5) | 2 (6) |
| OC [ng/mL] | t0 | 0 (0) | 0 (0) | 0 (0) | 0 (0) |
|  | t2 | 0 (0) | 1 (3) | 0 (0) | 0 (0) |
| cOC [ng/mL] | t0 | NA^c^ | | | |
|  | t2 |  |  |  |  |
| ucOC [ng/mL] | t0 | NA^c^ | | | |
|  | t2 |  |  |  |  |

RBP: retinol-binding protein, TTR: transthyretin, FAD: flavin adenine dinucleotide, HoloTC: holotranscobalamin, MMA: methylmalonic acid, Hcy: homocysteine, cB_12_: combined indicator, NA: not available, OC: total osteocalcin, cOC: carboxylated osteocalcin, ucOC: undercarboxylated osteocalcin.

Cells marked in grey indicate a deficiency.

a There is only a upper threshold for Hcy: <13 µmol/L

^b^ Reference range: -0.5-1.5 (vitamin B_12_ adequacy)

^c^ No reference range available for cOC and ucOC

**Supplemental Table S2** Frequency of consumption of animal products and other supplements within the study's allowed limits among participants

| **Variables [unit]** | **CON (n = 37)** | **INT (n = 35)** |
| --- | --- | --- |
|  | n (%) | |
| **Consumption of meat and/or meat products^a^** |  | |
| <1/month | 1 (2.7) | 2 (5.7) |
| Never | 36 (97.3) | 33 (94.3) |
| **Consumption of fish and/or fish products^a^** |  | |
| <1/month | 1 (2.7) | 1 (2.9) |
| Never | 36 (97.3) | 34 (97.1) |
| **Consumption of dairy products^a^** |  | |
| <1/month | 9 (24.3) | 10 (28.6) |
| 1-3/month | 3 (8.1) | 5 (14.3) |
| Never | 25 (67.6) | 20 (57.1) |
| **Consumption of eggs and/or egg products^a^** |  | |
| <1/month | 11 (29.7) | 7 (20.0) |
| 1-3/month | 1 (2.7) | 6 (17.1) |
| Never | 25 (67.6) | 22 (62.9) |
| **Use of nutrient supplements in general^b^** | 7 (18.9) | 7 (20.0) |
| Never | 30 (81.1) | 28 (80.0) |
| Vitamin B_12_ | 5 (13.5) | 5 (14.3) |
| Vitamin D_3_ | 3 (8.1) | 2 (5.7) |
| Vitamin K_2_ | 1 (2.7) | 2 (5.7) |
| Iron | 1 (2.7) | 0 (0.0) |
| Omega-3 | 1 (2.7) | 0 (0.0) |
| Other^c^ | 3 (8.1) | 2 (5.7) |
| No detailed information provided | 1 (2.7) | 1 (2.9) |

^a^ Including those in bakery products, pasta, or processed foods.

^b^ Multiple answers were possible

^c^ Turmeric, bases, coenzyme Q10, vitamin C, or resveratrol.

**Supplemental Table S3** Average nutrient biomarkers concentration at baseline (t0) and after 4 months (t2) and intragroup differences (in CON [n=37] or in INT [n=35]) in biomarkers concentration of the MultiVeg study participants from baseline to 4 months (t2-t0)

| **Nutrient biomarker [unit]** |  | **t0** | **t2** | **p-values^a^** |
| --- | --- | --- | --- | --- |
| Vitamin A [mg/L] | CON^b^ | 0.47 (0.20) | 0.68 (0.23) | **<0.001** |
|  | INT^b^ | 0.48 (0.14) | 0.69 (0.20) | **<0.001** |
| RBP [mg/dL] | CON^b^ | 1.90 (0.7) | 1.72 (0.53) | **<0.001** |
|  | INT^b^ | 2.00 (0.5) | 1.99 (0.53) | 1 |
| TTR [g/L] | CON^c^ | 0.26 ± 0.06 | 0.23 ± 0.05 | **<0.001** |
|  | INT^c^ | 0.25 ± 0.04 | 0.23 ± 0.05 | 1 |
| Beta-carotene [µg/L] | CON^b^ | 331.0 (427.0) | 416.0 (257.0) | 1 |
|  | INT^b^ | 409.0 (347.0) | 369.0 (307.0) | 1 |
| FAD [µg/L] | CON^b^ | 246.0 (43.0) | 196.0 (35.0) | **<0.001** |
|  | INT^b^ | 232.0 (31.0) | 209.0 (32.0) | **<0.001** |
| Vitamin B_12_ [pg/mL] | CON^b^ | 369.0 (266.0) | 293.0 (187.0) | **<0.001** |
|  | INT^b^ | 330.0 (204) | 376.0 (159.0) | 1 |
| HoloTC [pmol/L] | CON^b^ | 58.0 (37.0) | 72.0 (28.0) | 0.075 |
|  | INT^b^ | 50.0 (25.0) | 83.0 (20.0) | **<0.001** |
| MMA [µg/L] | CON^b^ | 18.0 (12.0) | 14.0 (6.0) | **<0.001** |
|  | INT^b^ | 20.0 (7.0) | 11.0 (5.0) | **<0.001** |
| Hcy [µmol/L] | CON^b^ | 10.0 (4.0) | 10.4 (4.3) | 0.548 |
|  | INT^b^ | 10.1 (5.0) | 9.2 (4.8) | 1 |
| cB_12_ | CON^c^ | 0.29 ± 0.58 | .34 ± 0.53 | 1 |
|  | INT^c^ | 0.17 ± 0.52 | 0.68 ± 0.45 | **<0.001** |
| Choline [µg/L] | CON^b^ | 746.0 (169.0) | 901.0 (238.0) | **0.011** |
|  | INT^b^ | 751.0 (182.0) | 940.0 (242.0) | **<0.001** |
| Folate [µg/L] | CON^b^ | 389.0 (82.0) | 332.0 (79.0) | **0.013** |
|  | INT^b^ | 376.0 (117.0) | 342.0 (123.0) | 0.672 |
| OC [ng/mL] | CON^c^ | 29.6 ± 9.8 | 28.2 ± 7.5 | 1 |
|  | INT^c^ | 32.0 ± 9.9 | 27.4 ± 8.6 | **0.014** |
| cOC [ng/mL] | CON^b^ | 11.4 (6.6) | 11.9 (6.2) | 1 |
|  | INT^b^ | 12.5 (5.1) | 11.7 (6.7) | 1 |
| ucOC [ng/mL] | CON^b^ | 10.6 (11.4) | 8.5 (6.1) | 1 |
|  | INT^b^ | 11.2 (8.4) | 7.6 (6.5) | **0.001** |

RBP: retinol-binding protein, TTR: transthyretin, FAD: flavin adenine dinucleotide, HoloTC: holotranscobalamin, MMA: methylmalonic acid, Hcy: homocysteine, cB_12_: combined indicator, OC: total osteocalcin, cOC: carboxylated osteocalcin, ucOC: undercarboxylated osteocalcin

^a^ Wilcoxon test (non-normally distributed continuous variables) or dependent t-test (normally distributed continuous variables), adjusted by Bonferroni-Holm correction.

^b^ Median (IQR). ^c^ Mean ± SD.
