# Supplementary figures and images for: Assessment of vitamin A, vitamin B2, vitamin B12, vitamin K, folate, and choline status following 4 months of multinutrient supplementation in healthy vegans: a randomised, double-blind, placebo-controlled trial
Source: Eur J Nutr. 2025 Dec 19;65(1):9. doi: 10.1007/s00394-025-03814-7 (PMC12717231; doi:10.1007/s00394-025-03814-7)

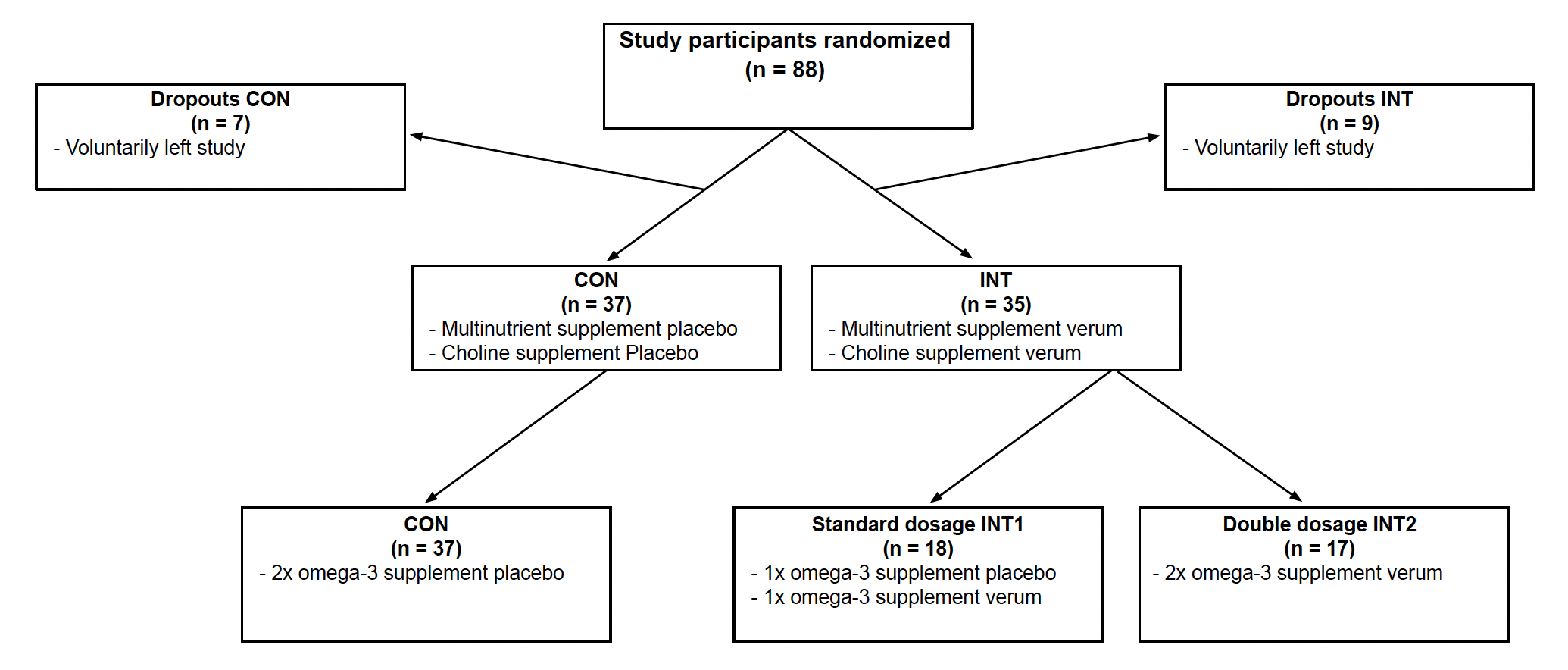

Supplement: Supplementary file 2 — Supplementary Material 2 [file 394_2025_3814_MOESM2_ESM.png]
